# Weighted cue integration for straight-line orientation

**DOI:** 10.1016/j.isci.2023.106072

**Published:** 2023-01-31

**Authors:** Shahrzad Shaverdian, Elin Dirlik, Robert Mitchell, Claudia Tocco, Barbara Webb, Marie Dacke

## Main text

(iScience *25*, 105207; October 21, 2022)

In the original article, in Equations 2 and 3 in STAR Methods, the parameters given in the estimators are rounded to two decimal places for brevity. The authors have found that the loss in precision caused by the rounding significantly affects the outcome of any calculations performed using these functions. This is an issue with the way the equations were written, not with the underlying code, which always uses the parameters at full precision. To this end, a disclaimer was added to the text following Equations 2 and 3:

"Note, line parameters given in Equations 2 and 3 are rounded for brevity and precision will significantly affect the outcome of the estimation. Parameters at full precision can be recovered from our codebase, or by using the same SciPy curve-fitting utilities on our precision data."

The authors apologize for any confusion this may have caused.

